# Coordinated gene expression during gilthead sea bream skeletogenesis and its disruption by nutritional hypervitaminosis A

**DOI:** 10.1186/1471-213X-11-7

**Published:** 2011-02-09

**Authors:** Ignacio Fernández, Maria Darias, Karl B Andree, David Mazurais, Jose Luís Zambonino-Infante, Enric Gisbert

**Affiliations:** 1 Unitat de Cultius Experimentals, IRTA Centre de Sant Carles de la Ràpita (IRTA-SCR), Crta. del Poble Nou s/n, 43540 - Sant Carles de la Ràpita (Spain; 2 Nutrition Aquaculture and Genomics Research Unit, UMR 1067, IFREMER Marine Fish Nutrition Team. IFREMER, Technopole Brest-Iroise, BP 70, 29280 Plouzané (France

## Abstract

**Background:**

Vitamin A (VA) has a key role in vertebrate morphogenesis, determining body patterning and growth through the control of cell proliferation and differentiation processes. VA regulates primary molecular pathways of those processes by the binding of its active metabolite (retinoic acid) to two types of specific nuclear receptors: retinoic acid receptors (RARs) and retinoid X receptors (RXRs), which promote transcription of downstream target genes. This process is well known in most of higher vertebrates; however, scarce information is available regarding fishes. Therefore, in order to gain further knowledge of fish larval development and its disruption by nutritional VA imbalance, the relative expression of some *RARs *and *RXRs*, as well as several genes involved in morpho- and skeletogenesis such as peroxisome proliferator-activated receptors (*PPARA*, *PPARB *and *PPARG*); retinol-binding protein (*RBP*); insulin-like growth factors I and II (*IGF1 *and *IGF2*, respectively); bone morphogenetic protein 2 (*Bmp2*); transforming growth factor β-1 (*TGFB1*); and genes encoding different extracellular matrix (ECM) proteins such as matrix Gla protein (*mgp*), osteocalcin (*bglap*), osteopontin (*SPP1*), secreted protein acidic and rich in cysteine (*SPARC*) and type I collagen α1 chain (*COL1A1*) have been studied in gilthead sea bream.

**Results:**

During gilthead sea bream larval development, specific expression profiles for each gene were tightly regulated during fish morphogenesis and correlated with specific morphogenetic events and tissue development. Dietary hypervitaminosis A during early larval development disrupted the normal gene expression profile for genes involved in RA signalling (*RARA*), VA homeostasis (*RBP*) and several genes encoding ECM proteins that are linked to skeletogenesis, such as *bglap *and *mgp*.

**Conclusions:**

Present data reflects the specific gene expression patterns of several genes involved in larval fish RA signalling and skeletogenesis; and how specific gene disruption induced by a nutritional VA imbalance underlie the skeletal deformities. Our results are of basic interest for fish VA signalling and point out some of the potential molecular players involved in fish skeletogenesis. Increased incidences of skeletal deformities in gilthead sea bream fed with hypervitaminosis A were the likely ultimate consequence of specific gene expression disruption at critical development stages.

## Background

Skeletogenesis is a critical process in vertebrates during which the skeleton develops in a genetically programmed manner, leading to normal anatomy that provides support and protection for the internal organs. In mammals, this process includes the differentiation and proliferation of different cell types, such as chondrocytes, osteoblasts, osteocytes and osteoclasts, which determine the size, shape and mineral composition of bone structures. The expression of specific genes deeply underlies these processes of cell proliferation and differentiation, which are also controlled by biotic and abiotic factors as well as individual genetic characteristics. Thus, determining factors and conditions that control and perturb those processes at the transcriptional level could be useful for deciphering the specific mechanisms behind these processes.

Teleost fish are considered to be the first vertebrate group to develop a bony skeleton, and with it, the molecular machinery necessary for its formation and maintenance. Thus, fish have been recognized as a suitable vertebrate model for understanding skeletogenesis in lower and higher vertebrates for both comparative [1] and evolutionary [2] purposes. In addition, marine fish larvae hatch much earlier in their development than other vertebrates, suggesting that the spatiotemporal sequences of the skeletal development in teleosts are quite different from those of higher vertebrates [3]. This makes marine fish species a very interesting model to study the influence of several nutrients, such as vitamin A, in morphogenesis and skeletogenesis during early larval development [4].

Nutritional research has recently focused on the role of nutrients on gene expression and regulation (nutrigenomics) [5]. The main pathway by which some nutrients control gene expression is by the activation of transcription factors, with the nuclear receptor superfamily being one of the most important. Within this family, the retinoic acid receptors (RARs) and retinoid X receptors (RXRs) are the specific receptors that transduce the vitamin A (VA) signalling by binding to their specific ligands, the retinoic acid isomers. RARs and RXRs have been found in all vertebrate tissues examined, and within both are present three different isotypes (α, β and γ), each one encoded by a separate gene. These RARs and RXRs bind to retinoic acid (RA, the main active metabolite of VA), becoming ligand-activated receptors. Then, they form homo- and/or heterodimers that bind to specific nucleotide sequences (retinoic acid response elements, RAREs) in the promoter region of a large number of genes [6,7], regulating their transcriptional activity. These RARs and RXRs can also affect indirectly the transcription of many other genes without any RARE in their promoter [6]. Then, through those molecular pathways, RA plays a key role in morphogenesis, cellular proliferation and differentiation processes of bone formation [8].

Skeletogenesis has been extensively described in several marine fish species [9-12], but gene expression patterns during larval development have only been partially characterized in European sea bass (*Dicentrarchus labrax*; [13,14]) and gilthead sea bream (*Sparus aurata*; [15]). In addition, studies have shown that fish fed diets with deficient or excess VA content had compromised development, showing reduced growth and survival rate, delayed digestive system maturation and high incidence of skeletal deformities [16-22]. However, while most reports are related to the consequences of dietary VA imbalance, limited work has focused on the molecular pathways involved in retinoid homeostasis that leads to an abnormal fish phenotype [23]. Moreover, such works have focused mainly on embryonic development rather than larval development [24-26]. Therefore, following our previous work on the description of abnormal phenotypes in gilthead sea bream larvae fed with hypervitaminosis A [18], in the present study we evaluated the relative expression of several genes involved in gilthead sea bream morpho- and skeletogenesis during larval development, and their disruption when fish were fed with high VA doses. In addition to retinoic acid receptors (*RARA*, *RARG *and *RXRB*), we evaluated the expression of different genes interacting with the RA signalling pathway, such as peroxisome proliferator-activated receptors (*PPARA*, *PPARB *and *PPARG*) that act as regulators of lipid and lipoprotein metabolism, glucose homeostasis, cellular proliferation and differentiation, as well as apoptosis in mammals [27]; the gene encoding retinol-binding protein (*RBP*) as the main protein that specifically transports retinol from liver to peripheral tissues [28]; and insulin-like growth factors I and II (*IGF1 *and *IGF2*, respectively), which control cell growth and proliferation. Bone morphogenetic protein 2 (*Bmp2*) and transforming growth factor β-1 (*TGFB1*) were also studied as they are key transcriptional factors for normal development [29], controlling the production of different extracellular matrix (ECM) proteins of bone. Additionally, relative expression of genes encoding different ECM proteins was evaluated such as matrix Gla protein (*mgp*) and osteocalcin (*bglap*, also known as bone Gla protein) as important regulators of calcium metabolism and skeletal development; osteopontin (*SPP1*) and secreted protein acidic and rich in cysteine (*SPARC*), as examples of important matricellular proteins acting as modulators of ECM interactions; and type I collagen α1 chain (*COL1A1*), which represents 90% of collagen proteins in bone tissues. Present results, describing the expression of several genes involved in gilthead sea bream morpho- and skeletogenesis processes under standard larval rearing conditions and their disruption of expression under hypervitaminosis A regimens, are of basic interest for understanding normal skeletogenesis and the appearance of skeletal deformities, as well as a tool for assessing fish nutritional VA imbalance.

## Results

### Gilthead sea bream larval growth and bone mineralization

**Figure 1:**
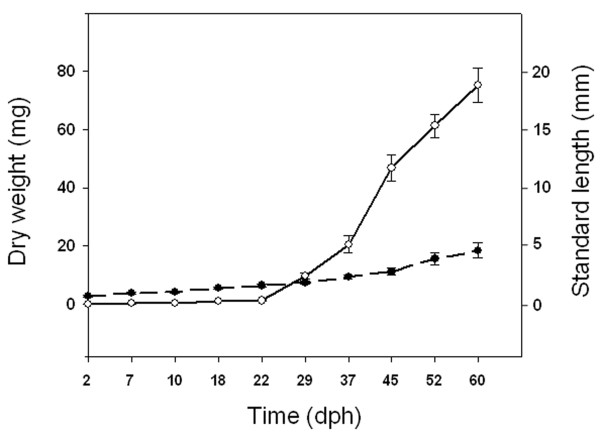
**Gilthead sea bream larval growth from 2 to 60 dph**. Growth in standard length (long dash line) was measured in mm and weight in mg dry weight (solid line). Values are expressed as mean ± standard deviation. Equation of the linear regression for the growth in standard length was y = 1,6562x - 0,666 (R^2 ^= 0,9112), while the exponential regression line for the growth in dry weight was y = 0,0252e^0,8815x ^(R^2 ^= 0,9614).

Larval growth in standard length (SL) and dry weight (DW) under standard rearing conditions are presented in Figure 1. Growth in DW and SL shows the typical exponential and linear increase with age, respectively. Growth performance of larval rearing under hypervitaminosis A conditions was affected by the level of dietary VA [18]. Whereas no clear effects were found in larval SL at 18 days post hatch (dph), larvae fed with the highest dietary VA content (10×VA; 10- fold VA increase in relation to the control diet) were significantly smaller (7%) than the control group at 60 dph. Similarly, no differences in DW were found between experimental groups at 18 dph, while at 60 dph 10×VA larvae (56.86 ± 11.27 mg) presented a reduction of 27% in DW with respect to control and 1.5×VA (1.5-fold VA increase in relation to the control diet) larvae (74.61 ± 8.84 and 69.31 ± 10.24 mg, respectively).

**Figure 2:**
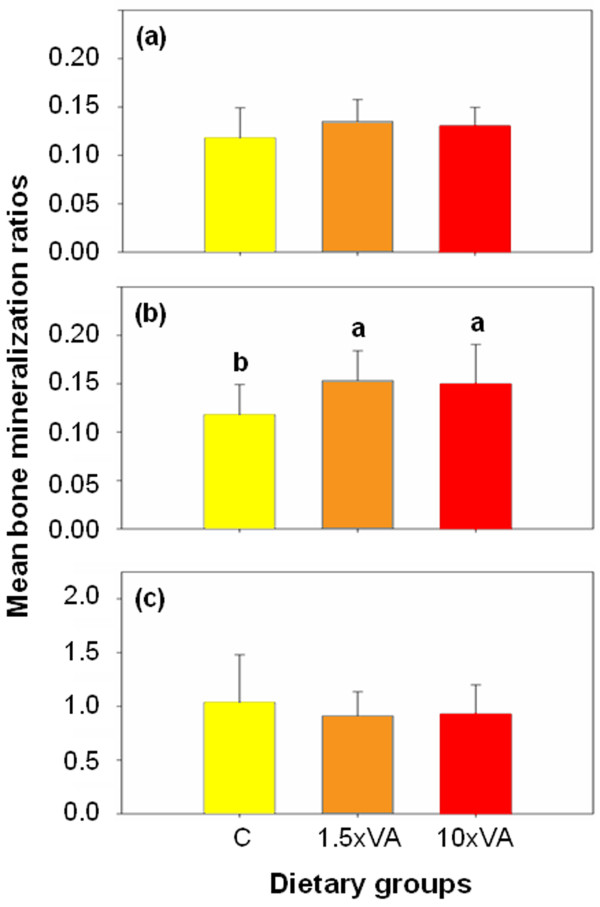
**Gilthead sea bream bone mineralization at 60 dph of fishes from the different dietary treatments**. Bone mineralization measured as ratios of red pixels (a), blue pixels per larval surface (b), and red pixels over blue pixels (c). Ratios are expressed as mean ± standard deviation. Letters denotes significant differences between dietary groups (ANOVA, *P *< 0.05; *n *= 26 larvae per treatment). C, larvae fed with control diet (0.66*10^8 ^total VA IU kg^-1 ^DW); 1.5×VA, larvae fed with 1.5 fold increase in dietary VA content (1.00*10^8 ^total VA IU kg^-1 ^DW); 10×VA, larvae fed with 10 fold increase in dietary VA content (6.82*10^8 ^total VA IU kg^-1 ^DW).

**Figure 3:**
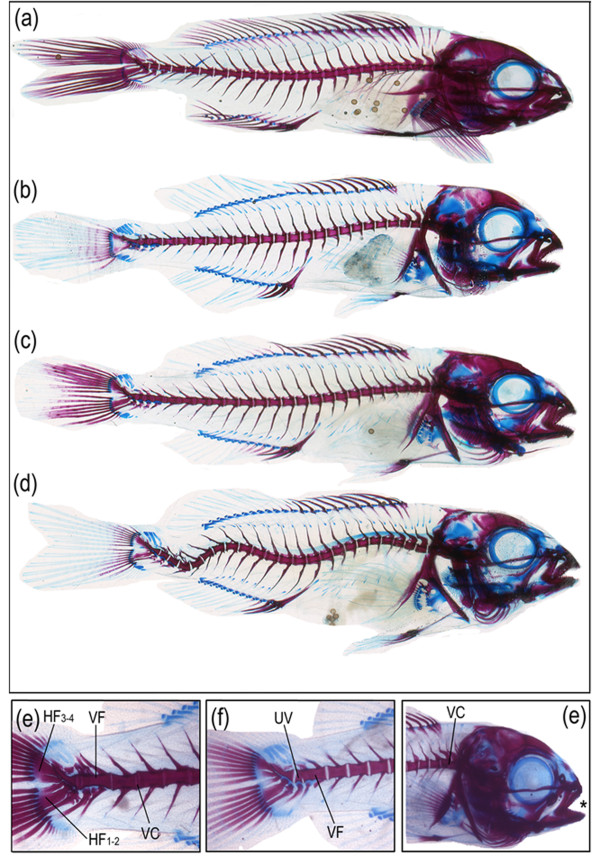
**Examples of double stained fish with alcian blue/alizarin red from different dietary treatments showing different levels of ossifications and typologies of skeletal deformities**. (a) Larva fed with control diet (0.66*10^8 ^total VA IU kg^-1 ^DW) exhibiting mostly red coloured skeletal structures (calcified bone) with the exception of pterygiophores and sclerotic elements still stained with alcian blue (cartilage). (b) Larva fed moderately increased levels of VA (1.5×VA group, 1.00*10^8 ^total VA IU kg^-1 ^DW) showing a larger proportion of skeletal structures stained in blue (cartilage) in comparison to the control group; *e.g. *pectoral fin girdle, epurals, parahypural and specialized neural arch in the caudal fin complex, and frontal, pterotic and parietal bones in the cranium. (c) Larva fed with the highest levels of VA (10×VA group, 6.82*10^8 ^total VA IU kg^-1 ^DW) showing intermediate values in blue colouration with regards to the control group. The skeletal structures stained in blue (cartilage) are those already reported in the larva from the 1.5×VA group. (d) Larva showing a severe deformity (double lordosis and kyphosis) affecting haemal vertebrae. (e) Deformed caudal fin complex showing the fusion of the hypurals 3-4 (HF3-4) and 1-2 (HF1-2), as well as the fusion (VF) and compression (VC) of different haemal vertebrae centra (23-24 and 22-23, respectively). (f) Fusion of vertebral bodies (VF) from haemal vertebrae number 22 and 23 and underdevelopment of vertebrae 24 (UV). (e) Head of a larva with a slight prognathism (asterisk) due to an underdevelopment of the premaxillar and maxillar bones, and vertebral compression (VC) between centra of prehaemal vertebrae number 2 and 3.

Regarding the bone mineralization of larvae, bone and cartilage staining quantification performed by IMAQ Vision Builder is summarized in Figure 2. Mineralization values were expressed as the ratio of red/blue colour as well as the specific amounts of each colour per larval surface, from stained fish for each experimental group. There were three patterns observed (Figure 3): (i) in control larvae most of the structures were red coloured with the exception of few structures (mainly pterygiophores and sclerotic related structures); (ii) the 1.5×VA larvae had many structures quite blue in colouration (pectoral and caudal fin related structures, pterygiophores, and splanchnocranium related structures such as frontral, pterotic, sphenotic and sclerotic related structures); and (iii) in the 10×VA larvae blue colouration was intermediate with respect to the previously enumerated structures of 1.5×VA larvae (skeletal structure nomenclature was as in [9-11]). Mineralization values in 1.5×VA and 10×VA larvae at 60 dph were higher than in the control group, although there were no significant differences (ANOVA, *P *> 0.05; Figure 2a). The absence of a statistically significant difference between treatments is likely due to the high variability observed among replicates from each treatment. Interestingly, significantly higher cartilage staining was found in 1.5×VA and 10×VA larvae with respect to the control group (ANOVA; *P *< 0.05; Figure 2b). In addition, the ratio of red/blue coloration (bone/cartilage mineralization) showed that both larvae fed with VA supplemented diets (1.5×VA and 10×VA) had lower values than the control group; although this tendency was not statistically significant (ANOVA, *P *> 0.05; Figure 2c).

### Gene expression patterns during gilthead sea bream ontogeny development under standard rearing conditions

**Figure 4:**
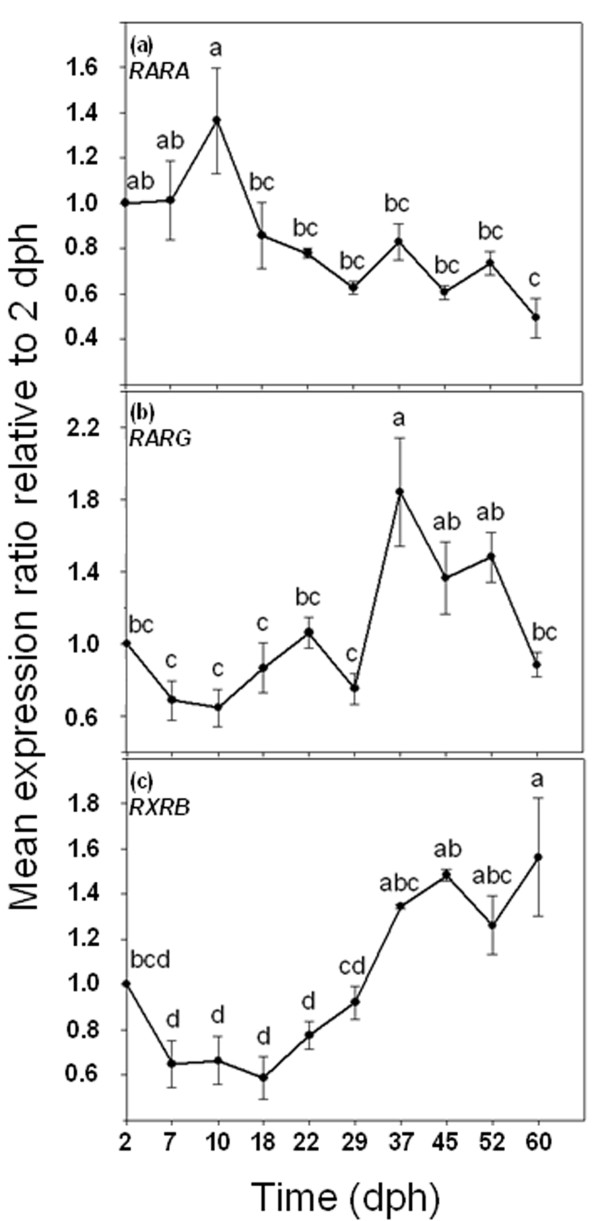
**Ontogenetic gene expression patterns of *RARA *(a), *RARG *(b) and *RXRB *(c)**. Gene expression measured as the mean expression ratio of the target gene with respect to the house-keeping gene (*EF1α*) at each sample time compared with initial sample time (2 dph). Different letters denote significant differences of the global gene expression (ANOVA, *P *< 0.05; *n *= 3).

During larval development, RA nuclear receptors, *RARA*, *RARG *and *RXRB*, exhibited different expression patterns in gilthead sea bream (Figure 4). Under our rearing conditions, gilthead sea bream larvae showed a high overall level of *RARA *gene expression until 10 dph, after which levels decreased significantly and thereafter remained constant until the end of the experiment (60 dph) (ANOVA, *P *< 0.05; Figure 4a). Conversely, the expression of *RARG *and *RXRB *were lower during the initial larval development, after which the levels increased. However, while *RARG *increased significantly from 29 dph, with a peak at 37 dph (1.8 fold change compared to 2 dph), its expression level significantly decreased at 60 dph (ANOVA, *P *< 0.05; Figure 4b) with respect to the levels at 37 dph. The expression of *RXRB *increased significantly only between 29 and 60 dph (ANOVA, *P *< 0.05; Figure 4c).

**Figure 5:**
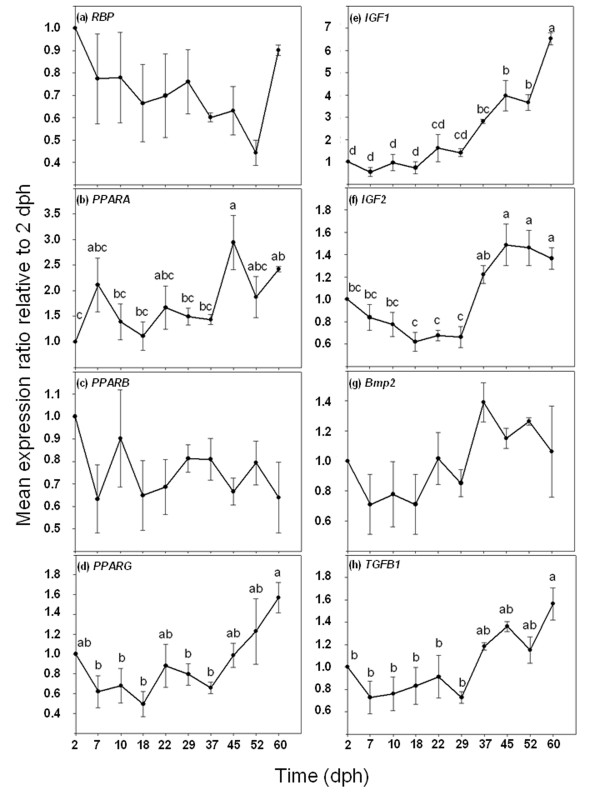
**Ontogenetic gene expression patterns of *RBP *(a), *PPARA *(b), *PPARB *(c), *PPARG *(d), *IGF1 *(e), *IGF2 *(f), *Bmp2 *(g), and *TGFB1 *(h)**. Gene expression measured as the mean expression ratio of the target gene with respect to the house-keeping gene (*EF1α*) at each sample time compared with initial sample time (2 dph). Different letters denote significant differences of the global gene expression (ANOVA, *P *< 0.05; *n *= 3).

There were no significant differences in the *RBP *gene expression profile during the larval development (ANOVA, *P *> 0.05; Figure 5a). *PPARA *expression was constant from 2 to 37 dph, whereas at 45 dph a peak of gene expression was noted (2.94 fold increase with respect to 2 dph larvae; *P *< 0.05; Figure 5b). *PPARB *expression remained constant from 2 to 60 dph (ANOVA, *P *> 0.05; Figure 5c). The *PPARG *expression level did not change from 2 to 52 dph; however, at the end of the study (60 dph), expression values were significantly higher than those mostly observed between 7 and 37 dph (ANOVA, *P *< 0.05; Figure 5d), but not significantly different to those observed at 2 dph.

Overall, the *IGF1 *and *IGF2 *gene expression had significantly increased at the end of larval development. However, while *IGF1 *gene expression increased significantly and progressively from 0.54 at 7 dph to 6.52 at 60 dph (ANOVA, *P *< 0.05; Figure 5e), *IGF2 *expression remained constant from 2 to 29 dph (ANOVA, *P *> 0.05; Figure 5f). Expression values of *IGF2 *increased significantly between 37 and 45 (1.43 ± 0.06 mean expression ratio; ANOVA, *P *< 0.05; Figure 5f) and remained constant until the end of the study. *Bmp2 *did not show significant variations in its mean gene expression ratio throughout larval development (ANOVA, *P *> 0.05), although a tendency for higher expression values was observed from 37 dph onwards (Figure 5g). Interestingly, *TGFB1 *presented an overall significant increase in its gene expression ratio from 29 dph until the end of the trial (ANOVA, *P *< 0.05; Figure 5h).

**Figure 6:**
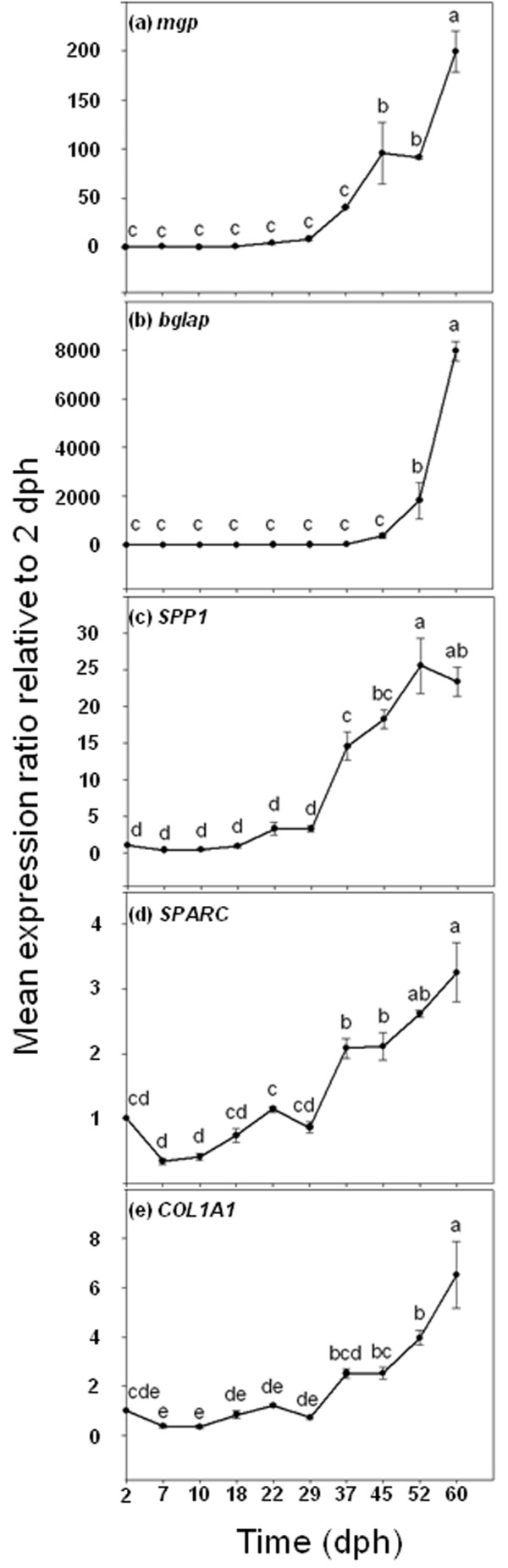
**Ontogenetic gene expression patterns of *mgp *(a), *bglap *(b), *SPP1 *(c), *SPARC *(d), and *COL1A1 *(e)**. Gene expression measured as the mean expression ratio of the target gene respect to the house-keeping gene (*EF1α*) at each sample time compared with initial sample time (2 dph). Different letters denote significant differences of the global gene expression (ANOVA, *P *< 0.05; *n *= 3).

With respect to the expression patterns of genes encoding ECM proteins during larval development, all of them increased at the end of the study. However, the mean gene expression ratios reached at the end of the experiment by each gene were different. The genes *mgp *and *bglap *showed the highest mean expression ratios at 60 dph (199.38 and 7956.15, respectively; ANOVA, *P *< 0.05; Figure 6a,b). In both Gla protein genes, a significant increase in the mean gene expression ratio was observed near the end of the trial (around 45 dph). In contrast, *SPP1 *and *SPARC *showed lower mean values (23.38 and 3.25, respectively) at the end of the experiment (Figure 6c and 6d, respectively) compared with *mgp *and *bglap *ratios, although presenting significant increases as well in comparison to results from early larval stages (ANOVA, *P *< 0.05). Furthermore, both *SPP1 *and *SPARC *showed a significant increase at 37 dph in their mean gene expression ratios, earlier than *mgp *and *bglap*. Finally, *COL1A1 *presented a similar gene expression profile to those of other ECM protein genes, with a significant increase in the mean gene expression ratio at 60 dph (ANOVA, *P *< 0.05; Figure 6e).

**Figure 7:**
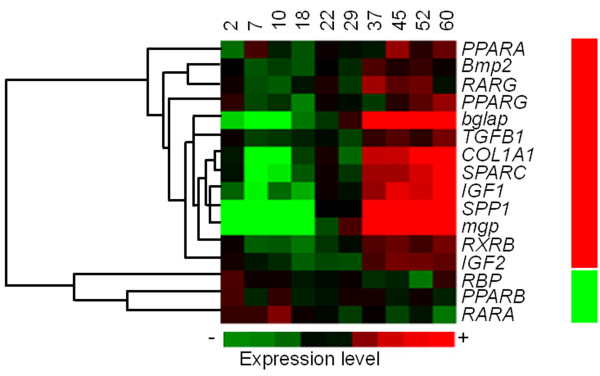
**Hierarchical clustering of the 16 target genes analysed based on mRNA expression, showing the cluster of the different studied genes with similar expression profile during larval development**. Columns represent the mean data values for each of the 10 sampling times (days post hatching; dph) and rows represent single genes. Expression level of each gene is represented relative to its median abundance across the different stages and is depicted by a colour scale: green, black, and red indicating low, medium, and high relative expression levels, respectively. Coloured bars to the right indicate the location of two gene clusters: red corresponds to genes with a progressive increase in mean gene expression level from 2 to 60 dph; and green with those whose mean gene expression level decreased progressively from an initial relative high level of expression.

Global hierarchical clustering was applied on the gene expression ratios during larval development for the 16 studied genes (Figure 7). This clustering was used to classify genes on the basis of similarity of their expression profile during larval development sampling times. Two gene clusters were found: (i) genes whose mean gene expression level increased from 37 dph, such as *IGF1*, *TGFB1*, *RXRB*, *IGF2 *and genes encoding extracellular matrix proteins (*mgp*, *bglap*, *SPP1*, *SPARC *and *COL1A1*); and (ii) genes whose mean gene expression ratio decreased progressively from an initial relatively high level of expression (*PPARB*, *RARA *and *RBP*).

### Gene expression in gilthead sea bream larvae fed with increasing levels of dietary VA

At 18 dph, larvae fed with increasing dietary VA levels exhibited disruption in the expression of several studied genes. While some of them showed an up-regulation, others were down-regulated with respect to the control feeding regime. That gene disruption was also noted in some genes even when the dietary VA imbalance had finished more than 40 days before (60 dph).

**Figure 8:**
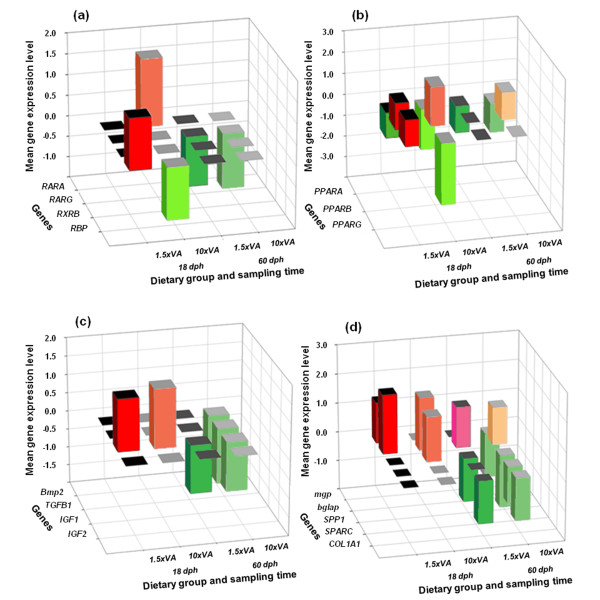
Relative gene expression of *RARA*, *RARB*, *RXRB *and *RBP*(a), *PPARA*, *PPARB *and *PPARG *(b), *Bmp2*, *TGFB1*, *IGF1 *and *IGF2 *(c) and *mgp*, *bglap*, *SPP1*, *SPARC *and *COL1A1 *(d) in fishes fed with experimental diets (1.5×VA and 10×VA, 1.00*10^8 ^and 6.82*10^8 ^total VA IU kg^-1 ^DW, respectively) at 18 and 60 dph. Relative gene expression measured as the fold change of the target gene with respect to the house-keeping gene (*EF1α*) at the appropriate sample time and compared with gene expression in the control group using REST 2008 software. Gene up- and down-regulations are highlighted in red and green (respectively), using different colour tone to identify each experimental group and sampled time. Only significantly higher or lower overall gene expression levels are represented (*P *< 0.05; *n *= 3 per dietary group).

Regarding RA nuclear receptors, *RARA *presented at 18 dph a significant up-regulation (1.6 fold change respect to the control group) in 10×VA larvae with respect to control fishes (REST, *P *< 0.05; Figure 8a); however no significant up- or down-regulation of *RARA *was found at 60 dph regardless of the dietary conditions (REST, *P *> 0.05; Figure 8a). In contrast, while no differences in *RARG *mean gene expression ratio were found at 18 dph in larvae fed with increased dietary VA content (1.5×VA and 10×VA), a slight but significant *RARG *down-regulation (1.2 and 1.32 fold change, respectively) was found with respect to control larvae at 60 dph (REST, *P *< 0.05; Figure 8a). Interestingly, no change in gene expression was found concerning the gene *RXRB *at both sample times analyzed (REST, *P *> 0.05; Figure 8a).

At 18 dph, an inverse correlation between increasing dietary VA and *RBP *gene expression was observed (Figure 8a). Larvae fed with a moderate increase in dietary VA content (1.5×VA) showed a significant up-regulation in the *RBP *gene expression ratio (1.23 fold change respect to the control group), whereas those animals fed with the highest levels of VA in diet (10×VA) presented a down-regulation with respect to control larvae (1.3 fold change; REST, *P *< 0.05; Figure 8a). However, no significant differences in *RBP *gene expression ratios were found at 60 dph between experimental groups (REST, *P *> 0.05; Figure 8a).

Expression levels of *PPARs *were also affected by dietary VA levels, however each one to a different extent (Figure 8b). Larvae fed with higher dietary levels of VA (1.5×VA and 10×VA) presented a significant down-regulation in *PPARA *at both sample times analyzed (18 and 60 dph); up to 1.9 fold change in 10×VA larvae at 18 dph compared with control larvae (REST, *P *< 0.05; Figure 8b). In contrast, larvae fed with higher VA diets showed a significant up-regulation in *PPARB *gene expression (up to 1.82 fold change in 10×VA 18 dph larvae) with respect to control larvae (REST, *P *< 0.05; Figure 8b), whereas no differences were observed in larvae from the 1.5×VA group at 60 dph. Interestingly, *PPARG *showed a contrary effect to the increase of VA dietary content at 18 dph. While there was a slight significant up-regulation in 1.5×VA larvae (1.2 fold change with respect to control fish; REST, *P *< 0.05; Figure 8b), 10×VA larvae exhibited a significant down-regulation (*ca*. 2.87 fold change with respect to the control group; REST, *P *< 0.05; Figure 8b). However, no differences in gene expression ratio were found in 1.5×VA and 10×VA larvae at 60 dph (REST, *P *> 0.05; Figure 8b).

At the end of the feeding phase with high dietary VA content (18 dph), *Bmp2 *and *TGFB1 *did not present significantly different gene expression ratios in larvae fed with supplemented VA diets (1.5×VA and 10×VA) compared with those from the control group (REST, *P *> 0.05; Figure 8c). This pattern was also observed at 60 dph in 1.5×VA larvae. However, *Bmp2 *and *TGFB1 *gene expression levels were significantly reduced with a 1.57 and 1.5 fold change (respectively) at 60 dph in larvae fed with the highest dietary VA content (10×VA) with respect to control larvae (REST, *P *< 0.05; Figure 8c). The *IGF *axis showed different responses to dietary VA content (Figure 8c). *IGF1 *gene expression was up-regulated in larvae fed with higher dietary VA levels at 18 dph (1.4 and 1.6 fold change in 1.5×VA and 10×VA, respectively) with respect to the control group (REST, *P *< 0.05); whereas a significant down-regulation at 60 dph (between 1.11 and 1.14 fold change) in *IGF1 *transcription was found with respect to the control group (REST, *P *< 0.05). Interestingly, *IGF2 *expression was not significantly different among experimental groups at both analyzed sampling times (REST, *P *> 0.05).

Genes encoding extracellular matrix (ECM) proteins were also differentially affected by dietary VA content (Figure 8d). The expression of *mgp *gene from 1.5×VA larvae at 18 dph was significantly up-regulated (1.39 fold change) compared with the control larvae (REST, *P *< 0.05), while non-significant differences were found in 10×VA larvae (REST, *P *> 0.05). Conversely, at 60 dph mRNA levels of *mgp *in 1.5×VA larvae was no significantly different with respect to the control group (REST, *P *> 0.05); whereas 10×VA larvae presented a significantly lower gene expression ratio (1.5 fold change) than the control larvae (REST, *P *> 0.05). Furthermore, the gene encoding the other Gla protein analyzed (*bglap*) showed significantly higher gene expression levels at 18 dph (2.02 and 1.81 fold change in 1.5×VA and 10×VA larvae, respectively) in groups fed with higher dietary VA content than the control (REST, *P *< 0.05). The effect of increasing dietary VA level at 60 dph was the same in 1.5×VA and 10×VA larvae. Fish from both treatments showed significant up-regulation on *bglap *expression (1.39 and 1.29 fold change) with respect to control larvae (REST, *P *< 0.05). In addition, when analyzing the expression of genes encoding matricellular proteins, *SPP1 *did not show significant differences in 1.5×VA larvae with respect to control larvae at 18 dph. However, a significantly higher gene expression ratio (1.54 fold change) was found in 10×VA larvae compared to the control group (REST, *P *< 0.05). Furthermore, *SPP1 *was down-regulated at 60 dph in both larvae fed with higher VA dietary content (1.48 and 1.78 fold change in 1.5×VA and 10×VA, respectively) with respect to the control group (REST, *P *> 0.05). Interestingly, *SPARC *only showed significant differences in expression in 10×VA larvae at 60 dph with respect to the control group (REST, *P *< 0.05), being lower by a 1.28 fold change. Finally, mRNA levels of *COL1A1 *were not affected by the dietary level of VA when compared to the experimental groups (REST, *P *> 0.05) at 18 dph. However, at 60 dph gene expression of *COL1A1 *was significantly lower in 1.5×VA and 10×VA larvae (1.45 fold change for both 1.5×VA and 10×VA groups) as compared to the control group (REST, *P *< 0.05).

## Discussion

The present study analysed the expression profile of several gene markers for skeletogenesis during the larval development in gilthead sea bream, and the influence of dietary hypervitaminosis A on their expression. Reliable nutritional, physiological and gene expression results can be drawn from this work, as results in growth (dry weight and standard length) fell within the range of previously reported values [30,31]. Results from this study supported the idea that dietary VA content controlled normal fish development through binding of its active metabolite, retinoic acid (RA), with specific nuclear receptors RARs and RXRs, and regulated target genes expression levels including retinoic receptors themselves. However, as RNA extractions were obtained from pooled whole larvae, the expression profiles of each gene reflect the expression level from a mix of different cell types and tissues. Thus, the variation of gene expression ratios observed during larval development under standard conditions could reflect changes in proportions of different tissues throughout ontogenesis and/or gene expression regulation in a specific tissue. In this sense, two different types of gene expression profiles during gilthead sea bream larval development were found by hierarchical clustering, showing that transcription is time- and tissue-dependent for each gene. For example, it is evident that higher expression of genes encoding ECM proteins are correlated with body growth, as increased fish size requires increasing bone strength and skeleton size in order to support the increased body weight. As ECM genes are expressed only in specific tissues (*e.g. *skeleton), the disruption of their tissue and developmental stage dependent expression by VA could be inferred; whereas it would remain unclear for the other genes that are ubiquitously expressed (*e.g. IGFs*, *PPARs*, *Bmp2 *and *TGFB1*), since their disruption would be masked by the overall expression in other tissues.

### Retinoid Receptors

The different expression profiles of *RARA*, *RARG *and *RXRB *found in this study supported the hypothesis that each gene had a temporal and spatial specific expression; suggesting specific roles for each of them [13,32,33]; in which contrasted with the suggested redundancy among them as found in mouse null mutant studies [34]. Previous works pointed out that *RARA *plays a crucial role in vertebrate RA signalling, being ubiquitously expressed in embryonic and adult mammal [35,36] and fish tissues [13]. However, the reported evolution of *RARA *gene expression in this study is not in agreement with that found by Villeneuve and co-workers [13], where European sea bass *RARA *gene expression increased from 10 to 42 dph. It is possible that although both fish species are evolutionarily closely related, their timing of development is quite different, which may explain the above-mentioned differences. Interestingly, the fact that *RARA *is predominantly expressed at 5 dph in the jaws of European sea bass [13], supports the idea that high expression of *RARA *observed in 10×VA larvae may be specifically implicated in the development of jaw deformities detailed in our previous work [18]. Two factors would explain the high *RARA *gene expression found in 10×VA larvae compared to 1.5×VA and control larvae: (i) the transcriptional activation of *RARA *through the presence of a RARE in its promoter [7], is due to an increase in RA levels; and (ii) the increased gene expression of Cyp26 enzymes mediated through RARα receptor, in order to degrade the excessive RA into other metabolites [37]. Under normal conditions, chondrogenesis is accompanied by a decrease in *RARA *expression [38], a process that normally takes place at 10 dph in gilthead sea bream. This result supports the idea that the overexpression of *RARA *was dietary induced by an increase of VA, as reported in fish exposed to Am80 (a RAR-selective agonist [39]), RA [32] or hypervitaminosis A [16]. In addition, high transcription levels of *RARA *might be directly responsible for the disruption of normal patterns of skeletogenesis, and could be responsible for the high incidence of skeletal deformities found in fish fed with high levels of VA [18]. The *RARA *disruption might have also activated a downstream gene cascade, including Hoxd-4 and Shh [40] that would also have affected larval morphogenesis [41], and delaying the maturation of the digestive system as it was found at 18 dph larvae from the 10×VA group [18]. Furthermore, such differences in the development of the digestive system were not observed at 60 dph [18], when VA imbalance was corrected and *RARA *expression was normalized in relation to the control group.

The expression pattern of *RARG *during larval development suggests that this gene plays a crucial role in the transcriptional RA signal regulation during vertebrate morphogenesis, chondrogenesis and differentiation of squamous epithelia [42,43]. Our results show that the *RARG *gene expression ratio was highest between 37 and 52 dph, which is concomitant with the onset of the typical adult skin development in gilthead sea bream; and in concordance with that reported in European sea bass between 10-42 dph [13]. The modulation of *RARG *expression by hypervitaminosis A has been demonstrated in mammals [44] and fishes [16,17,32]. Nevertheless, no regulation of *RARG *expression by hypervitaminosis A was detected in gilthead sea bream larvae at 18 dph. This might be due to the fact that chondrogenesis in chondral bones is almost completed at that stage of development [9,11], and *RARG *is expressed at a lower level in hypertrophic than in pre-hypertrophic chondrocytes [45]. In contrast to 18 dph, larvae aged 60 dph exhibited a down-regulation of *RARG *in 1.5×VA and 10×VA groups, which under present experimental conditions seemed to be linked to different rates of larval development (e.g. chondrogenesis and skin differentiation) among dietary treatments.

The role of RXRs on retinoid signal transduction during development of vertebrates depends on each subtype. Despite null *RXRB *or *RXRG *mutant mice being viable, and do not display VA associated abnormalities, *RXRA *null mutants die [34]. In teleosts, RXRα is involved in the development of the anterior hindbrain, tailbud, neural crest, pharynx and fins; whereas RXRβ played different roles in early larval development; and RXRγ plays a key role in brain and nervous system development and function [26]. In the present study, the ontogenic increase in expression of *RXRB *during the standard experimental trial was in agreement with previously reported results [16], and it is related to the development of those tissues where *RXRB *is mainly expressed [26]. The expression levels of *RXRB *in gilthead sea bream larvae were not affected by high levels of dietary VA, which seems to confirm that all-*trans*-RA (the most abundant RA isomer in nature) does not bind to this nuclear receptor nor is it transcriptionally activated by RAREs [46].

In this study, we present data from gene expression at specific time points, which is a static representation of a dynamic process involving the formation of homo- and heterodimers of the translated proteins leading to downstream cascades of gene expression. Variations in RAR-RXR homo-/heterodimer equilibrium have been shown to cause severe abnormalities in zebrafish embryos [47]. Thus, abnormal skeletogenesis and/or morphogenesis in gilthead sea bream could be interpreted as a perturbation of the nuclear RAR-RXR homo-/heterodimer equilibrium, as higher amount of *RARA *transcripts could induce increased formation of RARα-RXR heterodimers or decreased formation of RXR heterodimers with its different partners (VDR, TR, PPARs, etc).

### Retinol-Binding Protein

Retinol Binding Protein (RBP) is reputed for transporting retinol from the liver to different target tissues [48]. In agreement with previous studies in fish [48] and other vertebrates [49], *RBP *gene expression was low during larval development, and remained constant until metamorphosis. Such low *RBP *expression might be due to either low retinol mobilization requirements during early fish larval development, or to the fact that the daily requirements for VA were already fulfilled. Therefore, retinol mobilization from liver to target tissues was not needed, and consequently the *RBP *not transcribed. When gilthead sea bream larvae were fed with moderately increased levels of VA (1.5×VA group) at 18 dph, *RBP *expression was up-regulated, whereas this gene was down-regulated in fish fed with the highest levels of VA (10×VA group). Those changes in *RBP *gene expression by the dietary VA levels may be due to transcriptional regulation through the double RARE in its promoter. Considering that VA homeostasis via its release from storage tissues is a tightly controlled process [50], we suggest that the observed *RBP *gene expression in gilthead sea bream larvae in response to dietary VA content is part of a protective mechanism to avoid VA toxicity. Under slight dietary hypervitaminosis A (1.5×VA group) conditions, an increase in *RBP *mRNA level could be directly induced by binding of ligand activated RARs and RXRs to its RARE [51]; whereas during exposure to elevated VA levels (10×VA group), a decrease in *RBP *expression might take place to reduce the mobilization of VA from the adipose tissue and liver [52].

### Peroxisome proliferator-activated receptors

The peroxisome proliferator-activated receptors (PPARs) are well known fatty acid and eicosanoid inducible nuclear receptors in vertebrates, playing multiple physiological functions [53]. Molecular studies recently showed that there exist differences in tissue expression and ligand-binding properties between fish and mammalian PPARs [54-56].

Considering that PPARα is implicated in the regulation of fatty acid metabolism [27], the progressive increase in *PPARA *expression at late larval stages seemed to be correlated to larval growth and progressive differentiation of the liver, intestine and muscle, where this receptor is mainly expressed [54,57]. However, *PPARA *expression was down-regulated when larvae were exposed to high levels of dietary VA at 18 dph, which might be linked to the impaired maturation of the digestive system of 1.5×VA and 10×VA larvae in comparison to the control group [18]. This is in agreement with [54], *PPARA *expression being dependant on the nutritional status of the animal and its changing energy requirements during development. As PPARα plays an important role in adipocyte differentiation in fishes [56], its down-regulation in gilthead sea bream early juveniles fed with hypervitaminosis A might have also perturbed the normal differentiation rate of mesenchymal cells into myogenic, osteogenic and/or adipocytic cells, leading to skeletal deformities.

PPARβ in mammals is involved in the skeletal, brain and skin functions as well as in adipose tissue differentiation and fatty acid metabolism [58,59]. Its early expression in gilthead sea bream might be linked to the mobilization of endogenous reserves stored in the yolk sac [54] and to the synthesis and turnover of cellular membranes [60]. However, it did not correlate significantly with growth or fat deposition, as it has been previously reported in cobia (*Rachycentron canadum*) [57]. Increased expression of *PPARB *has been described in the early phase of adipogenesis in mammals [61] and red sea bream (*Pagrus major*) [56]; but in contrast to mammalian *PPARB*, its expression in adipocytes did not seem to be under nutritional control. Primary osteoblastic cells showed a high expression of *PPARB *[59], then the higher gene expression of *PPARB *in 1.5×VA and 10×VA larvae compared to the control group at 18 dph might indicate a premature osteoblastogenesis in those groups, as has been suggested in our previous study [18]. Interestingly, it has been reported that muscle-specific overexpression of *PPARB *in mice resulted in a profound change in muscle fibre composition due to hyperplasia [62]. Impaired muscle development could in turn induce some vertebral deformities [63] and lead to those skeletal deformities (*e.g. *lordosis) reported in our previous study [18]. These changes in muscle were coupled with a reduction in the mass of body fat of mice [62] that may be in agreement with the gene expression of the above-mentioned *PPARA *and *PPARG*. In addition, *PPARB *was up-regulated in 10×VA larvae at 60 dph, which could reflect a retarded adipogenesis. It is clear that more research is needed to reveal the role of PPARβ in regulating the muscle fibre growth and the adiposity of marine fish, as well as the potential induction of skeletal deformities by such impaired muscle development.

In mammalians, PPARγ regulates adipogenesis at its early phase through heterodimerization with RXR [64]. In fish, it seems that PPARγ fulfil the same roles, although they are not activated by the same specific ligands [54]. Under normal rearing conditions, *PPARG *expression increased with gilthead sea bream ontogenesis similarly to data reported in cobia where *PPARG *expression increased with fish growth and fat deposition [57]. However, it has been reported that retinaldehyde (the metabolic precursor of RA) inhibits *PPARG*, resulting in a lower PPAR-RXR complex formation [65], which in turn could enhance osteogenesis instead of adipogenesis in mesenchymal cells. Thus, lower *PPARG *expression in fish fed with high levels of VA (10×VA group) at 18 dph could reflect an enhancement of osteogenesis and disrupt normal skeletogenesis in gilthead sea bream larvae. This hypothesis is also reinforced by the increased blue coloured surfaces (chondrocytes) in both 1.5×VA and 10×VA larvae compared with control larvae.

### Growth Factors

Skeletal cells synthesize different growth factors, such as fibroblast growth factor, platelet-derived growth factor, IGFs, TGFβ, and additional cytokines. The expression of *IGF1 *is found in different soft and calcified tissues in adult gilthead sea bream [66], where three spliced variants with a specific pattern of expression have been found [67,68]. Our Taqman assay was designed to recognise all three *IGF1 *splice variants; therefore, our reported gene expression ratio for *IGF1 *is the sum of the expression for all three splice variants. In the present study, the progressive increase of *IGF1 *expression was in accordance with the high cell proliferation rate, and/or the increase in specific cell activity in different tissues during larval morphogenesis [66]. The dose-dependent overexpression of *IGF1 *found at 18 dph in gilthead sea bream larvae fed hypervitaminosis A, was in agreement with previous works [16,69]. The higher *IGF1 *expression in the 10×VA group might be due to synergistic direct and indirect effects. The growth hormone promoter contains a RARE [70], and has been found to be regulated by RARα/γ isoforms in pituitary cells of carp (*Cyprinus carpio*; [71]). In turn, increased *RARA *expression might induce growth hormone transcription and secretion, which finally would induce an increase in *IGF1 *hepatic transcripts. In addition, increased levels of thyroxin (T_4_) were observed in fish under hypervitaminosis A [19], while T_4 _is reported to induces *IGF1 *expression in *in vitro *fish studies [72]. Then, it seems plausible that the increase in expression of *IGF1 *in gilthead sea bream larvae fed with high VA levels (1.5×VA and 10×VA groups) could be due also to increased T_4 _levels. Such high *IGF1 *expression, which is known to promote muscle differentiation and growth [73], coupled with that of *PPARB*, might have caused an imbalance in the development of the musculoskeletal system in gilthead sea bream fed with high levels of VA, leading to a higher incidence of lordosis in those larvae [18].

In the present study, the abrupt increase in *IGF2 *expression recorded at the end of the larval phase (29 dph) corresponds with the onset of ossification in most of the bone structures [9,11], which is mainly due to its role in osteoblast proliferation and differentiation [74]. In contrast to *IGF1*, the expression of *IGF2 *in gilthead sea bream fed with hypervitaminosis A was not disrupted, supporting the idea that different hormonal signals and mechanisms of gene transcription control the regulation of expression of both *IGF *forms [67].

Other growth factors such as transforming growth factors beta (TGFβs) or the bone morphogenetic proteins (BMPs) are important for the development of bone, among other tissues [29]. Among those BMP's, BMP-2 plays a key role in bone development, inducing the differentiation of mesenchymal cells into osteoblast precursors and promoting the maturation of osteoblasts through the expression of *Runx2*/*Cbfa1 *[75]. TGFβ1 is involved in the regulation of a broad range of biological processes, including cell proliferation, differentiation and migration, production of extracellular matrix [76], as well as maintaining bone homeostasis and turnover [77]. In our study, the gene expression profile of *Bmp2 *in larvae reared under standard conditions seemed to correlate with the biological function of BMP2. *Bmp2 *expression between 18 and 22 dph might be associated with the onset of pre-osteoblasts proliferation [9,11]; whereas the observed tendency of *Bmp2 *to increase between 29 and 37 dph might be due to its role as a promoter of osteoblast differentiation. Furthermore, increased expression of *TGFB1 *was concomitant with an increase in expression of several genes encoding ECM proteins, as has been reported [76].

*Bmp2 *expression in early larval stages (18 dph) fed with hypervitaminosis A was not affected. However, the possibility cannot be neglected that in those larvae RA might have disrupted *Bmp2 *expression prior to our sampling point, as has been shown *in vitro *studies [33]. In contrast, *Bmp2 *was down-regulated in 10×VA larvae at 60 dph, suggesting a negatively controlled regulation of *Bmp2 *[78]. This down-regulation of *Bmp2 *concomitant with lower *RARG *gene expression is in accordance with the reported loss of the RA-inducible expression of *Bmp2 *in the absence of *RARG *gene expression [33]. In addition, the *Bmp2 *down-regulation might down-regulate the expression of ECM encoding genes (*mgp*, *SPP1*, *SPARC*, *COL1A1*) observed in 10×VA larvae through the transcriptional regulation of Runx2/Cbfa1 [78].

BMP-2 and TGFβ1 have opposing actions on osteoblast function and differentiation. While BMP-2 enhances *Runx2*/*Cbfa1 *expression, TGFβ1 inhibits its expression; then, both genes regulate the coordinated expression of several genes encoding ECM proteins [78]. The fact that both genes (*Bmp2 *and *TGFB1*) were down-regulated in gilthead sea bream larvae fed with hypervitaminosis A (10×VA group) at 60 dph, could be explained by the ubiquitous expression of *TGFB1 *which had a variety of other biological functions and therefore, this down-regulation of *TGFB1 *may not be representative of the skeletal tissue alone.

### Genes encoding bone extracellular matrix proteins

Until the beginning of the ossification process at 18 dph (5.7-6.0 mm standard length; [9], gene expression profiles of the genes encoding ECM proteins (*mgp*, *bglap*, *SPP1*, *SPARC *and *COL1A1*) showed low gene expression values. These results were in accordance with the ongoing development of most skeletal structures, that were not yet ossified, with the exception of some located in the viscerocranial and caudal region [9-11]. Higher gene expression was found from 37 dph onwards, concomitantly with the intense ossification of most of the bone structures, such as vertebrae centra (9.0-9.4 mm standard length; [9]). The expression of ECM proteins progressively increased during larval ontogeny. However, the significant increases of each gene occurred at different developmental times, the first to increase being *SPARC *and *SPP1*, followed by *mgp*, and finally by that of *COL1A1 *and *bglap*, which shows the progression of gene transcription of specific bone matrix development markers.

The early increase in expression of *SPARC *during gilthead sea bream ontogenesis reflects the many key processes during early larval development in which that protein is involved, showing an enhanced expression in areas undergoing chondrogenesis, osteogenesis, somitogenesis and angiogenesis [79]. SPARC is also reputed for inhibiting adipogenesis and enhancing osteoblastogenesis and fibrogenesis in rainbow trout [80], as well as for participating in the final mineralization and remodelling of the ECM. Our increase in *SPARC *expression during gilthead sea bream larval development is in agreement with that reported in rainbow trout (*Oncorhynchus mykiss*) [80], but was contrary to the results found in gilthead sea bream [79]. Although results are methodologically incompatible with those of [79] for comparative purposes, the increase in expression of *SPARC *during early larval ontogeny would seem more plausible, considering the participation of SPARC in many biological processes. Regarding *SPP1*, its early increased expression might be attributed to the differentiation of hypertrophic chondrocytes and osteoblasts [81].

In mammals, MGP is a decisive factor for differentiation and maturation of chondrocytes and a key regulator of chondral and intramembranous ossification [82]. In fish, branchial arches are the sites with higher levels of *mgp *expression, followed by the heart, vertebra, kidney and liver [83]. Thus, increased gene expression during larval ontogeny in our experiment could reflect the development of the above-mentioned organs and vital systems in order to match the biological needs of the developing larva.

*COL1A1 *is mainly expressed in connective tissues and is abundant in bone, cornea, and dermis, and in two cell types, osteoblasts and fibroblasts. Collagen fibres comprise 90% of the ECM proteins in skeletal tissues and confer most of their physical properties [84]. The present ontogeny of the *COL1A1 *expression was in agreement with previous results found in European sea bass, where *COL1A1 *was highly expressed from 31 dph onwards [14]. According to this data, the earlier increase in *COL1A1 *expression in comparison to that of *bglap *is in agreement with the temporal coordination of both ECM encoding genes [85]. Osteocalcin is a specific bone marker [86] that is required for the correct maturation of hydroxyapatite crystals during the process of calcification [87]. However, the relationship between osteocalcin and mineralization remains unclear even within the same species, since some authors detected *bglap *prior to mineralization and others at the onset or after the beginning of mineralization [82]. Its early detection at 2 dph, in contrast to the previous reported detection in gilthead sea bream at 37 dph [86], might be linked to the different molecular techniques used, since qPCR is more sensitive than Northern blot analysis. This earlier detection, prior to the development of mature osteoblasts and calcification processes, might be linked to its expression in chondrocytes undergoing chondral calcification, as reported in zebrafish [82]. However, the increase in *bglap *expression at older stages of development (52-60 dph) seemed to be due to the completion of the ossification of skeletal structures in the axial skeleton [9-11,18], similar to that found in European sea bass [88,89]. Considering that osteopontin and osteocalcin are involved in the modulation of hydroxyapatite crystallization [90], the advanced *SPP1 *expression might be related to the inhibition of osteoblast mineralization [91] occurring in vertebrae centra during their intramembranous ossification, thus allowing osteoblast to maintain their proliferative state; while *bglap *expression would be an indicator of the osteoblast mineralization in those structures [89].

The colour pixel analysis of skeletal structures revealed that bone development was affected by hypervitaminosis A, showing a disequilibrium between bone and cartilage, as 1.5×VA and 10×VA larvae display higher amounts of cartilage and lower values of red/blue coloration ratio with respect to the control group, respectively. Those lower ratios are in agreement with higher growth of some cartilage elements leading to the fusion of caudal fin complex structures [18]. Those differences in bone mineralization levels and ossification processes were reflected by changes in gene transcription, describing two different scenarios of mineralization stage for particular skeletal elements (splanchnocranium, dorsal and caudal fin elements) depending on the dietary VA level. Through its active metabolite RA, VA promotes terminal differentiation of hypertrophic chondrocytes [92], which could explain the higher amount of cartilage tissue in 1.5×VA and 10×VA larvae. Furthermore, *mgp *over-expression in 1.5×VA larvae at 18 dph indicated an abnormal development of cartilage, as *mgp *has been reported to control bone mineralization [93]. In contrast, high levels of VA in 10×VA larvae induced advancement of the mineralization process of chondral structures leading to a higher mineralization stage [18]. Such advanced mineralization could be explained by the down-regulation of *mgp *in 10×VA larvae at 60 dph and the higher expression of *SPP1 *at 18 dph compared to control and 1.5×VA larvae, as osteopontin is involved in osteoblast differentiation [91]. These results are in agreement with those reported in cell culture studies [94] and *in vivo *experiments [4]. At the end of the study, the down-regulation of *SPP1 *in 1.5×VA and 10×VA larvae might be considered as another sign of an abnormal osteoblast development, since SPP1 likely plays a key role in determining the biochemical properties of the bone [81]. In addition, another sign of the advancement of the mineralization process in skeletal structures in 10×VA larvae was the down-regulation of *SPAR*C at 60 dph.

Regarding *bglap *transcriptional regulation, it was up-regulated at 18 dph in 1.5×VA and 10×VA larval groups, reflecting an earlier chondral ossification of some skeletal elements derived from the splanchnocranium (*e.g. *maxillar, premaxillar, Meckel's cartilage, articular) which are the unique skeletal structures that were ossifying during that stage of development [11]. This precocious ossification process could be responsible for the high incidence of deformities in the above-mentioned structures described in our previous study [18]. Furthermore, at the end of the study (60 dph), fish fed with higher levels of VA (1.5×VA and 10×VA) showed a higher *bglap *expression than those from the control group. The over-expression of *bglap *in the fish fed hypervitaminosis A indicated an increased ongoing osteogenic processes, as those larvae presented a higher amount of osteogenic tissue (sum of red and blue coloured surfaces) compared to the control group. Consequently, present results suggest that if normal ranges for *bglap *expression are established, this gene might be a reliable marker for detecting disorders in bone formation and mineralization processes [95,89].

Finally, results of *COL1A1 *expression were not as informative as the other analyzed genes from the ECM regarding the skeletogenesis process. No changes in gene expression were found in *COL1A1 *in larvae fed with hypervitaminosis A at 18 dph compared to the control group, even though it has been shown that this gene contains a RARE in its promoter [6]. Different results from the effects of RA on the expression of *COL1A1 *are reported in the literature [6], which suggests an indirect regulation of *COL1A1 *by RA. However, larvae from the 1.5×VA and 10×VA groups showed lower expression of *COL1A1 *at 60 dph, which seems to be attributed to the lower expression of *RARG *detected in those animals, since this *RAR *binds specifically to the RARE of the *COL1A1 *promoter [84].

## Conclusions

The present study showed that the analysed gene expression patterns in the gilthead sea bream were correlated with skeletogenesis during early larval development, as they showed a temporally coordinated gene expression for specific markers of the ECM. Furthermore, results from this study supported the idea that perturbations in specific dietary nutrients can alter normal anatomic development mediated by specific genes controlled by ligand-receptor interactions. Although both experimental groups (1.5×VA and 10×VA) were fed with an excess of VA, there were differences in their juvenile phenotype depending on the degree of hypervitaminosis A. 1.5×VA and 10×VA larvae were fed respectively with moderate and high dietary VA content (1.5 and up to 10 fold increase of VA content in the diet with respect to the control group). From this we conclude, only hypervitaminosis A in the 10×VA group impaired larval performance in terms of growth, maturation of the digestive system and survival rate, in which the down-regulation of *RBP *is illustrative of excessive dietary VA. However, both doses of VA differentially affected the coordinated expression of genes during skeletogenesis, some of them being markers for the differences in the processes of chondrogenesis and osteoblastogenesis observed among dietary groups at early stages of larval development (i.e., *PPARG *and *mgp *for 1.5×VA; *RARA *and *SPP1 *for 10×VA). In particular, the up-regulation of *bglap *in 1.5×VA and 10×VA larvae indicated an advanced ossification in some skeletal structures. Early disturbance of skeletogenesis were still manifest after 40 days, as revealed by differences in gene expression, highlighting the importance of a good nutritional balance during larval development that determines juvenile phenotype.

Concluding, present results showed that fish are reliable animal models to study the effects of nutritional hypervitaminosis A. However, since only global effects of dietary VA on fish larval physiology could be inferred from this study due to the use of whole organisms, *in vitro *research with bone cell lines is needed to understand the mechanisms by which RA controls skeletogenesis.

## Methods

### Larval rearing and diets

Gilthead sea bream larvae (1 dph) were obtained from a Spanish private hatchery and shipped to the IRTA facilities. After their acclimation, larvae were distributed at an initial density of 100 larvae L^-1 ^in 2 and 24 cylindrical tanks, of 500 and 100 L respectively, connected to a water recirculation unit. Water conditions were as follows: 18-19°C, 35 ppt salinity, pH 7.8-8.2. Water was provided with gentle aeration and oxygenation (> 4 mg l^-1^) and 20% was exchanged daily. Photoperiod was 12L:12 D, and light intensity of 500 lux at water surface. All animal experimental procedures were conducted in compliance with the experimental research protocol approved by the Committee of Ethic and Animal Experimentation of the IRTA (reference number 621303898-3898-4-8), which followed the international principles of replacement, reduction and refinement for the use of animals in research.

On one hand, in order to characterize gene expression patterns of selected genes during larval development until the juvenile stage, larvae kept in 500 L cylindrical tanks were reared following a commercial production procedure. Feeding schedule was as follows: from day 4 to 20 post hatch (dph) rotifers (*Brachionus plicatilis*), whose density was progressively increased from 5 to 10 rotifers mL^-1^; *Artemia *nauplii (EG, INVE, Belgium) from 16 to 22 dph, in increasing density from 0.5 to 2 nauplii mL^-1^, and 2 days enriched-metanauplii from 20 to 40 dph (1 to 5 metanauplii mL^-1^). Both live preys were enriched with Easy Selco (ES; INVE, Belgium) according to manufacturer's instructions. From 36 dph to the end of the experiment (60 dph), larvae were progressively weaned onto dry feed, first with Proton 1/2 and 1/4 (INVE, Belgium) and then with Gemma Micro (size range: 75 to 500 μm; Skretting, Spain). On the other hand, larvae kept in 100 L tanks were reared in order to evaluate the effects of high dietary VA content on gilthead sea bream larval performance and quality ([18], as well as skeletogenesis-related gene expression (present work). Feeding sequence and dietary experimental conditions are described in detail in [18]. In brief, three different dietary regimes (each one in triplicate) were evaluated during the early larval development (rotifer feeding phase), containing graded levels of VA. The graded VA levels in live prey were obtained by adding retinol palmitate (1,600,000 IU g^-1^, Sigma-Aldrich, Spain) to the commercial enriching emulsion, Easy Selco™(ES, INVE, Belgium). Those dietary treatments are referred to as Control, 1.5×VA and 10×VA; and contained a mean of 0.66*10^8^, 1.00*10^8 ^and 6.82*10^8 ^total VA IU kg^-1 ^DW in enriched rotifers [18], while mean total vitamin A content in larvae was 60157, 71617 and 72909 IU g^-1 ^DW at 18 dph respectively.

### Sample collection

Biological samples were taken at 2, 7, 10, 18, 22, 29, 37, 45, 52 and 60 dph, in order to establish the gene expression patterns of skeletogenesis-related genes and larval growth during larval development. In order to evaluate gene expression regulation by hypervitaminosis A and bone mineralization, larvae reared under hypervitaminosis A were sampled at 18 dph, coinciding with the end of the nutritional challenge with VA (end of rotifer-feeding phase) and the onset of mineralization of the skeleton [9-11], and the end of the weaning period (60 dph). In all cases, larvae were sacrificed with an overdose of anaesthetic (Tricaine methanesulfonate, MS-222, Sigma). In both experiments, samples were frozen in RNA later (Ambion^®^) and stored at -80°C until gene expression analysis.

### Larval growth and bone mineralization

For larval growth, sampled larvae (*n *= 15) from each tank were washed with distilled water to avoid marine salts and used for body length and dry weight determination. Larval standard length was measured with a digital camera connected to a binocular microscope Nikon SMZ 800, AnalySIS (Soft Imaging Systems, GmbH). Once larvae lengths were measured, they were dried at 60°C until their weight was constant. Weights were obtained with an analytic microbalance Sartorius BP211 D. Thirty larvae per dietary group (ten larvae per tank) were stained with alcian blue and alizarin red S [18] in order to evaluate the level of bone mineralization under different nutritional circumstances and quantified as described in [22], using a computerized image analysis package (IMAQ Vision Builder, National Instruments, Austin, TX). Total red and blue pixels were considered as a marker of the relative ratio of osteoblasts and chondrocytes (respectively) and normalized by larvae body surface, as larval size were highly variable within tanks.

### RNA extraction and qPCR conditions

Total RNA was extracted from pools of fish larvae (100 to 5 individuals per sample time and tank depending of fish size) using the TRIzol reagent (Invitrogen^®^, San Diego, CA, USA) as specified by the manufacturer. The quantity of RNA isolated was determined using a GeneQuant spectrophotometer (Amersham Biosciences), measuring optical density at 260 nm and its purity was established by the absorbance ratio 260/280 nm (1.7-2.0). The quality of the RNA was examined using 1.2% agarose gel electrophoresis. A reverse transcription reaction was carried out using equal quantities of total RNA (1 μg) from each sample and Quanti Tect Reverse Transcription Kit (Qiagen^®^). Electrophoresis using a 1.2% agarose gel was run to assess the RT-PCR product.

Real-time qPCR was performed using an ABI PRISM 7300 (Applied Biosystems). For each gene, a species-specific Taqman assay was designed (Applied Biosystems) using the sequences acquired from the GenBank database (Table 1). The efficiency of the Taqman assay for each gene was previously evaluated to assure that it was close to 100%. All reactions were performed in 96 well plates in triplicate in 20 μl reaction volumes containing: 10 μl of 2× TaqMan universal PCR master mix (Applied Biosystems); 1 μl of the 20× Taqman primer/probe solution corresponding to the analyzed gene; 8 μl of molecular biology grade water; and 1 μl of cDNA diluted 1:10, with the exception of *bglap*, which was evaluated with straight dilution. Standard amplification parameters were as follows: 95°C for 10 min, followed by 45 amplification cycles, each of which comprised 95°C for 15 s and 60°C for 1 min. Real time qPCR was performed for each gene, and therefore, a calibrator sample was included within each plate.

**Table 1:** Accession number, primers and probes used for relative quantification of gene expression during gilthead sea bream ontogenic development and dietary vitamin A nutritional imbalance.

Gene name	Genebank	Component	5' to 3' nucleotide sequences
*RARA*	EU643830	Forward	CCTGTCTGGACATCCTGATACTTC
		Reverse	CGTGAGTCCATCTGAGAAAGTCAT
		FAM probe	CTCTGGTGTGTAGCGTGTAC

*RARG*	EU643831	Forward	GTGCGTAATGACAGAAACAAGAAGA
		Reverse	ACTCCTCTAGCTCTCCACTTAGC
		FAM probe	CTTTCTGGAAGCACCACCTC

*RXRB*	AM980430	Forward	CCTGAGGCCCATGCAATCTC
		Reverse	ACACACATGCGTTTCTGAGACAA
		FAM probe	CAGCCCTGGACTAATG

*PPARA*	AY590299	Forward	CTTTTCGTGGCTGCCATTATCTG
		Reverse	CTCCACCAAAGGCACATCCA
		FAM probe	CCTGGGCGATCTCC

*PPARB*	AY590301	Forward	GTTTGTTGCTGCCATCATTCTCT
		Reverse	CACCTGCTTCACGTTCATTAGC
		FAM probe	CCGGGACGATCTCCAC

*PPARG*	AY590304	Forward	CAATGTCGGCATGTCACACAAC
		Reverse	CTCCTTCTCCGCCTGGG
		FAM probe	CCGGCCAAAACGAATG

*IGF1*	AY996779	Forward	GGGCGAGCCCAGAGA
		Reverse	GCCGTAGCCAGGTTTACTGAAATAA
		FAM probe	TCCACACACAAACTGC

*IGF2*	AY996778	Forward	GTCGGCCACCTCTCTACAG
		Reverse	TGCTTCCTTGAGACTTCCTGTTTT
		FAM probe	TTACCCGTGATGCCCC

*Bmp2*	AY500244	Forward	GTGGCTTCCATCGTATCAACATTTT
		Reverse	GCTCCCCGCCATGAGT
		FAM probe	CAGGAGCTCCAAATAA

*TGFB1*	AF424703	Forward	TTTTCCAACTTCGGCTGTACTGT
		Reverse	GAGATGCCAAAACTGAAGGTACTGA
		FAM probe	ATTGCGGCCGTTCTAG

*RBP*	AY550957	Forward	TGGCCACCTTCGAGACAAC
		Reverse	GATGCGGCTCCCCAGTAG
		FAM probe	CCCCGCCAAGTTCAG

*mgp*	AY065652	Forward	CGCCCGAAATACACCTCAGA
		Reverse	GACGGACGGATACTAGGAGTCTA
		FAM probe	ACCAGCCGCGACTCG

*bglap*	AF048703	Forward	CGAGCACATGATGGACACTGA
		Reverse	GTCCGTAGTAGGCCGTGTAG
		FAM probe	CAGCGATGATTCCC

*COL1A1*	DQ324363	Forward	GGCAACAGTCGCTTCACCTA
		Reverse	CCCCATGTACCGGTGTGT
		FAM probe	ACGTGCATCCATCCTC

*SPP1*	AY651247	Forward	CCAGCCAGGAGTCAGAGGAT
		Reverse	ACTCTCATCTGAGTCGCTGTCA
		FAM probe	CTGCTCTGGCTCTCC

*SPARC*	AJ564190	Forward	AAGCTGCACCTCGACTACATC
		Reverse	CCTTCAGCTCGCTGTCCAT
		FAM probe	CAGGGCTCGATCATTT

*EF1α*	AF184170	Forward	CCCGGTATGGTTGTCACCTT
		Reverse	GGTGCATCTCCACAGACTTGAC
		FAM probe	CCCCAGCTGACCACTG

*18S*		TaqMan^® ^Gene Expression Assay\Eukaryotic 18S rRNA, 20X (Part Number 4331182)	

*18S *accession number, forward and reverse primers, as well as probe sequences are not shown as the Taqman assay used is proprietary.

### Data processing

The relative gene expression ratio for each gene was based on the PCR efficiency (E) and Ct of a sample compared with the control, and expressed in comparison to the reference gene, according to Pfaffl's mathematical model:

$$Ratio=\frac{E\text{ target gene (dCt target gene }(\text{control}-\text{sample})\text{)}}{E\text{ endogenous control gene (dCt }EF1\alpha \text{ RNA (control}-\text{sample))}}$$

Statistical differences in gene expression between different developmental stages in larval rearing under standard conditions were analyzed by ANOVA, while differences in gene expression ratios between dietary treatments at each sampled time in larval rearing under hypervitaminosis A were performed by randomization tests using REST 2008 software [96].

As reference genes, *18S *and *EF1α *were evaluated as those have been suggested to be reliable house-keeping genes for qPCR analyses of developmental processes [97]. However, results were finally evaluated only with *EF1α *as the reference gene, due to the low efficiency of the *18S *Taqman probe (< 90%). In the Control group, target and housekeeping gene expressions were measured at 2, 7, 10, 18, 22, 29, 37, 45, 52 and 60 dph using gene expression at 2 dph as the reference time point, to establish the normal ontogeny of target gene expression; while in the excess dietary VA groups (1.5×VA and 10×VA) the relative gene expression of the same target genes was evaluated at 18 and 60 dph, using the control group gene expression as the reference.

A supervised hierarchical clustering was applied [98] to the samples from the larval rearing under standard conditions and each gene was classified according to its gene expression profile. Tree View software, was used to generate visual representations of the classification [98].

In order to represent gene expression of target genes in a more comprehensive manner, gene expression ratios in larval rearing under hypervitaminosis A were reported as fold change regulation, and then ratios between 0 and 1 were transformed and represented as -1/(target gene ratio).

## List of abbreviations

*bglap*: osteocalcin or bone Gla protein gene; BMPs: bone morphogenetic proteins; BMP-2: bone morphogenetic protein 2; *Bmp2*: bone morphogenetic protein 2 gene; Cbfa1: core binding factor α 1 protein; *Cbfa1*: core binding factor α 1 gene; *COL1A1*: type I collagen α1 chain gene: Ct: Cycle threshold; dl: decilitre; dph: days post hatch; DW: dry weight; E: efficiency; ECM: extracellular matrix proteins; *EF1α*: elongation factor 1 α gene; GH: growth hormone protein; *GH*: growth hormone gene; Gla: y-carboxyglutamic acid; IGF-I: insulin-like growth factor I protein; *IGF1: *insulin-like growth factor I gene; IGF-II: insulin-like growth factor II protein; *IGF2: *insulin-like growth factor II gene; MGP: matrix Gla protein; *mgp*: matrix Gla protein gene; mRNA: messenger ribonucleic acid; PPARα: peroxisome proliferator-activated receptor α protein; *PPARA*: peroxisome proliferator-activated receptor α gene; PPARβ: peroxisome proliferator-activated receptor β protein; *PPARB*: peroxisome proliferator-activated receptor β gene; PPAR γ: peroxisome proliferator-activated receptor γ protein; *PPARG *: peroxisome proliferator-activated receptor γ gene; qPCR: semiquantitative polymerase chain reaction; RA: retinoic acid; RARα: retinoic acid receptor α protein; *RARA*: retinoic acid receptor α gene;RARβ: retinoic acid receptor β protein; *RARB*: retinoic acid receptor β gene; RARE: retinoic acid response element; RARγ: retinoic acid receptor γ protein; *RARG*: retinoic acid receptor γ gene; RBP: retinol binding protein; *RBP*: retinol binding protein gene; REST: Relative expression software tool; RNA: ribonucleic acid; RXRα: retinoid × receptor α protein; *RXRA*: retinoid × receptor α gene; RXRβ: retinoid × receptor β protein; *RXRB*: retinoid × receptor β gene; RXRγ: retinoid × receptor γ protein; *RXRG*: retinoid × receptor γ gene; Runx2: runt related transcription factor 2 protein; *shh*: Sonic hedge hog gene; SL: standard length; SPARC: secreted protein acidic and rich in cysteine; *SPARC*: secreted protein acidic and rich in cysteine gene; *SPP1*: osteopontin gene; T_4_: thyroxin; TGFβ1: transforming growth factor β 1 protein; *TGFB1*: transforming growth factor β 1 gene; TR: thyroid receptor; VA: vitamin A; VDR: vitamin D receptor;

## Authors' contributions

IF participated in the design of the nutritional dose-response experiment and conducted it, carried out the gene expression analyses and drafted the manuscript. MD participated in the gene expression analyses and in the final redaction of the manuscript. KA designed and optimized gene expression analyses and participated in the final redaction of the manuscript. DM and JZ participated in the gene expression analysis and in the final redaction of the manuscript. EG got the funding, conceived part of the study, participated in the experimental design and in the final redaction of the manuscript. All authors read and approved the final manuscript.
